# Postprandial changes of oxidative stress biomarkers in healthy individuals

**DOI:** 10.3389/fnut.2022.1007304

**Published:** 2022-09-30

**Authors:** Fengyi Huang, Xue Shen, Yuzheng Zhang, Ann M. Vuong, Shuman Yang

**Affiliations:** ^1^Department of Epidemiology and Biostatistics, School of Public Health, Jilin University, Changchun, China; ^2^Department of Epidemiology and Biostatistics, School of Public Health, University of Nevada, Las Vegas, NV, United States

**Keywords:** oxidative stress biomarkers, regular diet, fluorescent oxidation products, malondialdehyde, total antioxidant capacity, superoxide dismutase

## Abstract

Food consumption induces oxidative stress in humans, but the changes in oxidative stress levels after a regular meal are still unclear. We conducted an experimental study on 20 healthy volunteers (10 males, 10 females), who matched in age (±2 years). They were given a regular diet (total energy of 704 kcal, which contains 75 g of carbohydrates, 35 g of protein, and 29 g of lipids) at 11:30 a.m. after a fast of over 12 h. We collected 6-repeated measures of venous blood samples at 2-h intervals *via* heparin anticoagulant tubes immediately after the meal (indicated as “0” h) and up to 10 h post-consumption. Biomarkers included plasma fluorescent products, plasma malondialdehyde, plasma total antioxidant capacity, and plasma superoxide dismutase. FlOPs were measured at three excitation/emission wavelengths (FlOP_320, FlOP_360, and FlOP_400). The average age and BMI for the twenty participants were 22.70 ± 1.98 years and 20.67 ± 2.34 kg/m^2^, respectively. Within 10 h after the meal, the overall trend of FlOPs were generally similar. There was no evidence of dose response for any of the three FlOPs (all *P* > 0.05). However, levels of MDA decreased with the time of fasting (*P*_linear_ and *P*_quadratic_ < 0.05), with the biggest decrease occurring between 0 and 2 h post-meal. The overall trend of T-AOC and SOD levels also decreased with fasting time (*P*_linear_ and *P*_quadratic_ < 0.05), though an increase was observed between 0 and 2 h following consumption. Levels of MDA, T-AOC, and SOD but not FlOPs, decreased with fasting time.

## Introduction

Oxidative stress is characterized as an insufficient endogenous antioxidant defense system against the overproduction of reactive oxygen species (ROSs) (1). A high level of oxidative stress causes damage to lipids, proteins, and DNA, which leads to an increased risk of many chronic conditions, such as cardiovascular disease, cancer, chronic kidney disease, and osteoporosis (2–6). The level of oxidative stress in the body can be measured by a variety of biomarkers, such as fluorescent oxidation products (FlOPs), malondialdehyde (MDA), total antioxidant capacity (T-AOC), and Superoxide dismutase (SOD). FlOPs are stable and sensitive oxidative stress biomarkers that reflect the oxidation damage from proteins, lipids, and DNA (7, 8). MDA is one of the final products of cell polyunsaturated fatty acid peroxidation. It is widely recognized as one of the most sensitive markers to assess lipid oxidative damage (9). T-AOC is a good indicator of diet quality reflecting the antioxidant capacity of a diet, and it refers to the ability to predict plasma antioxidant status (10). SOD has an antioxidant function and its mechanism of action is mainly to scavenge the harmful superoxide anion radical. It is considered to be an efficient defense step to protect organisms from negative impacts (11).

Many studies suggests that oxidative stress is influenced by different diets in the human body (12, 13). There is evidence indicating that a diet high in fat, animal-based proteins and carbohydrate induces oxidative stress (14–16). In contrast, an antioxidant enriched meal or supplementation (i.e., vegetables and fruit) is able to reduce levels of oxidative stress in the human body (17–19). To date, few studies have examined the impact of a regular diet on oxidative stress (20). In addition, the specific timing for accurately measuring oxidative stress biomarkers following a regular diet is still unclear. This study aimed to examine the postprandial changes of FlOPs, MDA, T-AOC, and SOD in healthy individuals following a regular diet. Understanding the postprandial changes of oxidative stress may help prevent food-induced oxidative damage. Further, this study will help refine the timing of blood sample collection for accurately measuring oxidative stress levels in humans.

## Materials and methods

### Study setting and subjects

Twenty healthy volunteers (10 males, 10 females) were enrolled in the study. All males were individually matched with a female participant by age (±2 years). The study protocol was approved by the Institute of Research Board of the School of Public Health, Jilin University (Project #: 2018-03-05 and project #: 2021-12-06). Written informed consent was obtained from all individual participants.

### Dietary intervention

Participants were instructed to refrain from consuming food and drinks after 23:00 p.m. the day before the trial. On the day of the trial, all subjects were given a regular meal at 11:30 a.m. The meal mainly consisted of 75 g of carbohydrates, 35 g of protein, 29 g of lipids; its total energy was about of 704 kcal. The observational period lasted for 10 h. During the experiment, no additional meals or calorie food were allowed. Water was allowed *ad libitum*.

### Blood sample collection and storage

Venous blood samples were drawn every 2 h *via* heparin anticoagulant tubes, more specifically, immediately after the meal (indicated as “0” h), the 2nd hour, the 4th hour, the 6th hour, the 8th hour, and the 10th hour. These tubes were processed and stored in a −80°C refrigerator until completion of the oxidative stress biomarkers' assay.

### Measurement of oxidative stress biomarkers

Levels of FlOPs in plasma were measured by a modified Shimasaki's method, which was previously applied by our previous study (2). In brief, we extracted plasma with ethanol/ether (3:1, v/v) and with supernatant added for the detecting instrument. The 96-well Microplate (96-well Black Flat Bottom Polystyrene High Bind Microplate 3925, Corning^®^, USA) and a fluorescent microplate reader (Cytation3 Cell Imaging Multi-Mode Reader, BioTek, Vermont, USA) were used to determine the fluorescence. We measured fluorescence of the supernatant at three wavelengths, including FlOP_320 (excitation 320 nm, emission 420 nm), FlOP_360 (excitation 360 nm, emission 420 nm), and FlOP_400 (excitation 400 nm, emission 475 nm). FlOP_320 represents the interaction of lipid oxidative products, particularly lineolate, with DNA and metals. FlOP_360 represents the interaction between lipid oxidation productions and protein, DNA, and carbohydrates. FlOP_400 represents the interaction between MDA, proteins, and phospholipids. The level of fluorescence was expressed as relative fluorescent intensity units per milliliter of plasma. The within-run mean coefficients of variation (CVs) for the measurements at all three wavelengths were < 3.6%.

Plasma MDA was detected according to the guideline of the human MDA Elisa kits (Nanjing Jiancheng Bioengineering Institute, Nanjing, Jiangsu, China). The levels of MDA were determined by the spectrophotometric method at 532 nm after boiling the sample and condensing it with thiobarbituric acid (TBA).

The levels of T-AOC were determined through Erel's automated method, using the total antioxidant capacity assay kit (Nanjing Jiancheng Bioengineering Institute, Nanjing, Jiangsu, China). It is generally based on the loss of the characteristic color of a stable 2,2′-azinobis-(3-ethylbenzthiazoline-6-sulfonic acid) (ABTS) radical cation by antioxidants. The final results are expressed as micromoles of Trolox equivalents per liter.

Plasma superoxide dismutase (SOD) was measured among 6 males and 6 age matched females using the human SOD Elisa kits (Nanjing Jiancheng Bioengineering Institute, Nanjing, Jiangsu, China). The levels of SOD were determined by the spectrophotometrically method at 450 nm.

### Statistical analysis

The normality of the data was analyzed with the Shapiro–Wilk test. We used repeated measures analysis of variance (ANOVA) to assess the differences in postprandial oxidative stress biomarkers between different time points. A two-sided *P* ≤ 0.05 was identified to be statistically significant. All analyses were performed with the SPSS statistical software (Version 24.0, IBM SPSS, IBM Corp, Armonk, NY, USA).

## Results

**Figure 1:**
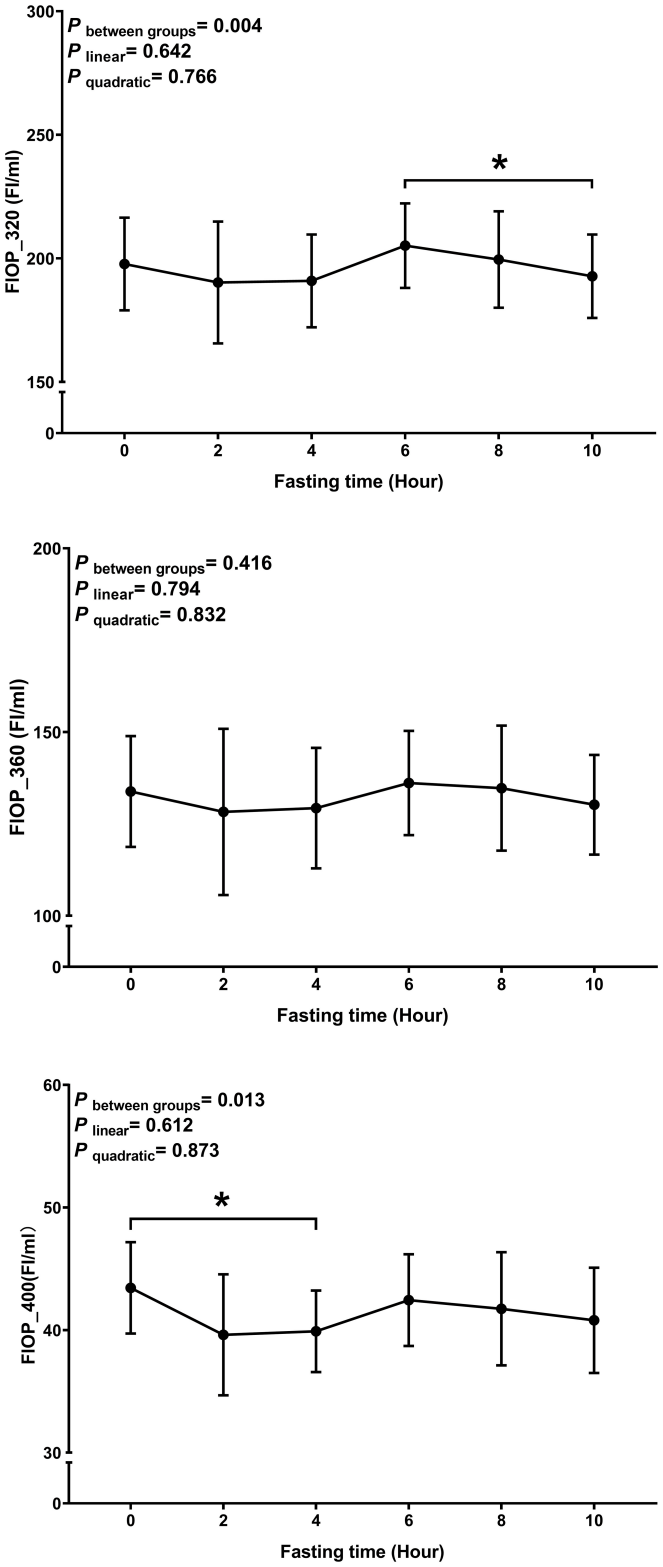
Postprandial changes in plasma FlOPs with fasting time. Data are presented as the means, with error bars representing the 95% confidence intervals of the means. *Indicates a statistically significant difference of FlOPs at the 10th hour as compared with the other time points at alpha = 0.05; all statistically significant differences are displayed.

The average age and BMI of the study participants were 22.70 ± 1.98 years and 20.67 ± 2.34 kg/m^2^, respectively. Postprandial changes of plasma FlOPs are shown in Figure 1. The overall trend of FlOP_320, FlOP_360, and FlOP_400 was generally similar within 10 h after the meal. There was no evidence to suggest that any of the FlOPs under the three wavelengths had a significant trend (all *P*_linear_ and *P*_quadratic_ > 0.05). However, we did observe statistically significant differences amongst FlOP_320 and FlOP_400 between post-meal measurements.

**Figure 2:**
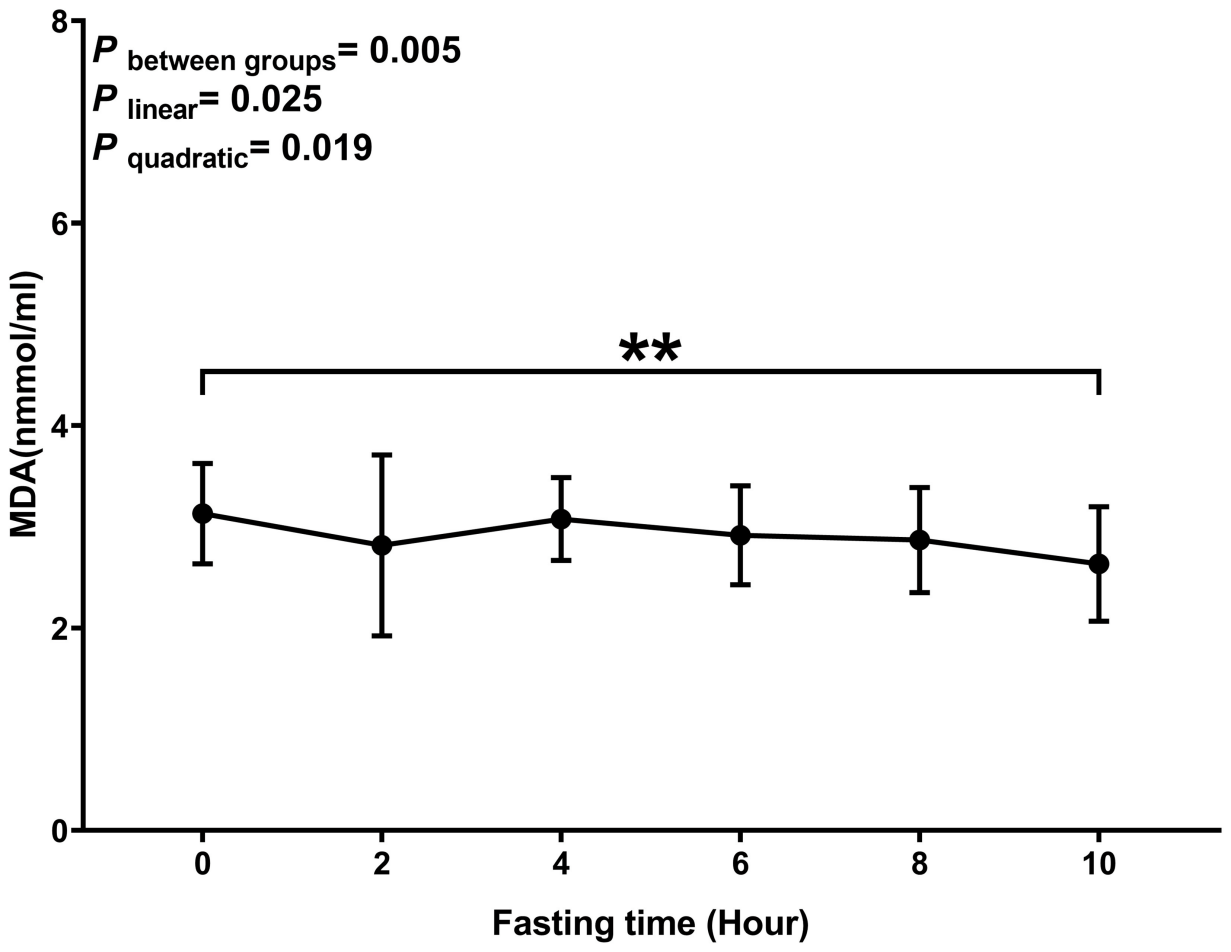
Postprandial changes in plasma MDA. Data are presented as the means, with the error bars representing the 95% confidence intervals of the means. **Indicates a statistically significant difference of MDA at the 10th hour as compared with the other time points at alpha = 0.01; all statistically significant differences are displayed.

Levels of MDA decreased with the time of fasting (*P*_linear_ and *P*_quadratic_ < 0.05) (Figure 2). The biggest decrease was observed within 2 h after the meal.

**Figure 3:**
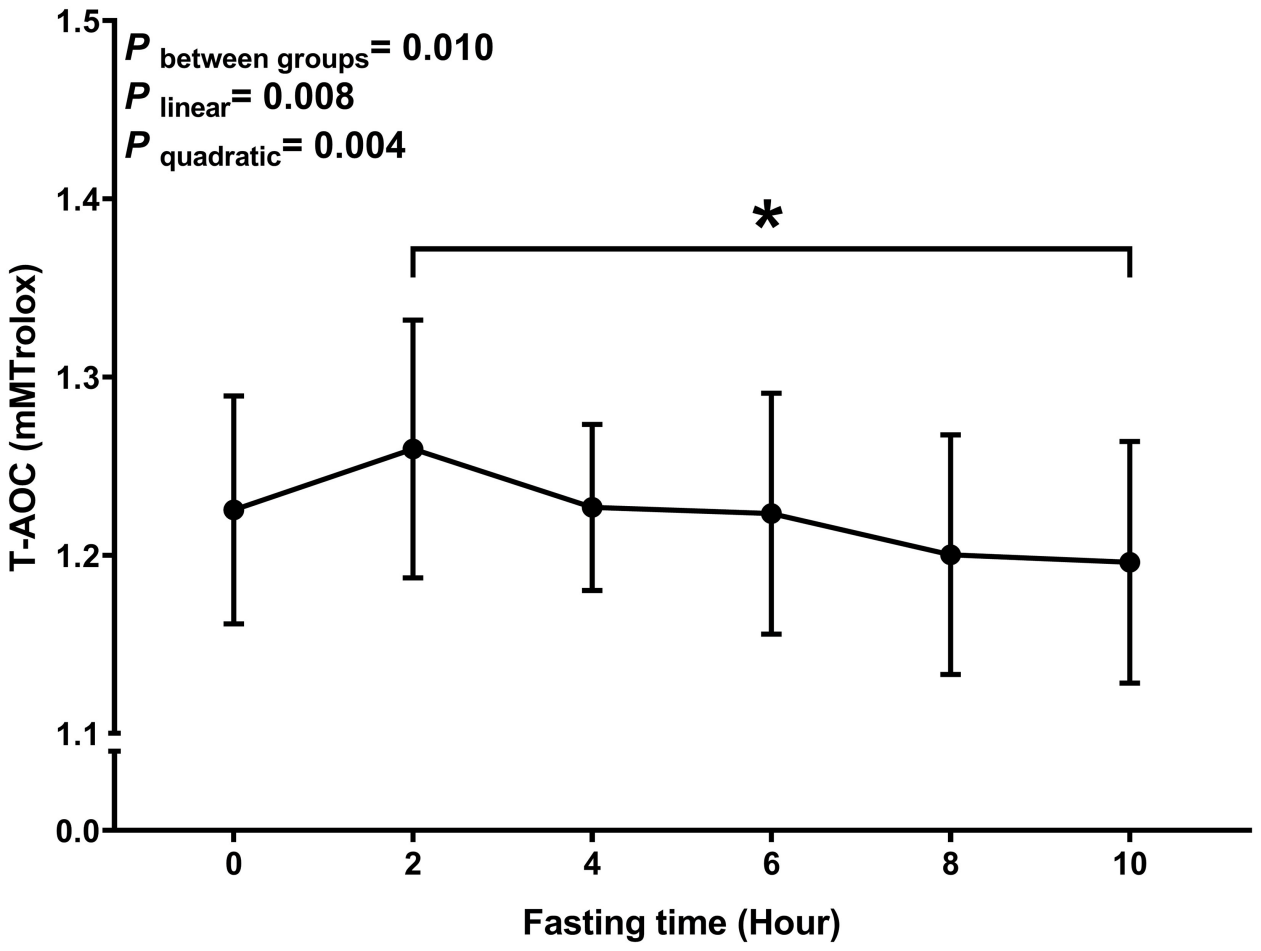
Postprandial changes in plasma T-AOC. Data are presented as the means, with the error bars representing the 95% confidence intervals of the means. *Indicates a statistically significant difference of T-AOC at the 10th hour as compared with the other time points at alpha = 0.05; all statistically significant differences are displayed.

**Figure 4:**
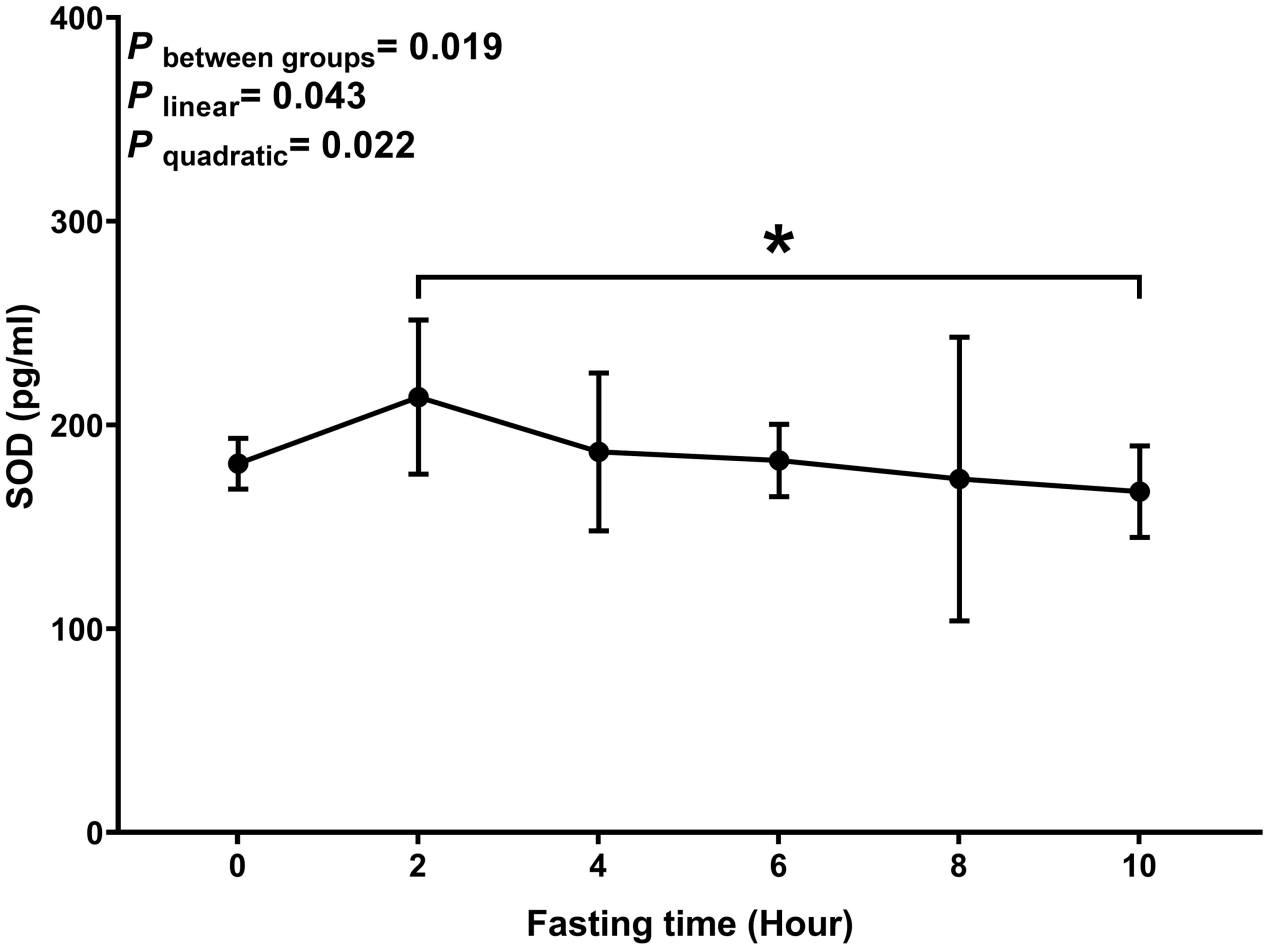
Postprandial changes in plasma SOD. Data are presented as the means, with the error bars representing the 95% confidence intervals of the means. *Indicates a statistically significant difference of SOD at the 10th hour as compared with the other time points at alpha = 0.05; all statistically significant differences are displayed.

Similar to MDA, we observed a decreasing trend with T-AOC and SOD levels with fasting time (*P*_linear_ and *P*_quadratic_ < 0.05) (Figures 3, 4). In contrast, there was an increase in T-AOC and SOD levels between 0 and 2 h post-consumption. However, their levels gradually decreased after 2 h of meal consumption.

## Discussion

In this experimental study of healthy individuals, we observed postprandial changes of FlOPs, MDA, T-AOC and SOD after a regular meal. We found that MDA, T-AOC and SOD levels, but not FlOPs, decreased as meal consumption time progressed. T-AOC levels and SOD levels increased between 0 and 2 fasting hours, then decreased onward.

Few studies have examined postprandial changes of oxidative stress in humans following a regular diet (15, 20–23). A previous cross-sectional study found that the relationship of different diets on FlOP levels in the body was slight (21), which is partly consistent with our findings. These findings suggest that FlOPs may not be a good biomarker for detecting the effects of a regular diet on the level of oxidative stress in the humans. However, FlOP_360 may be a suitable oxidative stress biomarker when fasting blood samples are unable to be obtained.

MDA is one of the products formed by the reaction of lipids with oxygen free radicals and its level represents the level of lipid peroxidation. The overall decreased trend in postprandial MDA levels with fasting time in our study can be explained by the decreased lipid absorption with longer fasting time in the body. This is in line with a previous study (15), in which MDA levels decreased following a lipid, carbohydrate, protein mixed meal. Interestingly, we also observed a decreased trend of MDA levels from 0 to 2 h of fasting and increased trend of MDA levels from 2 to 4 h of fasting following a regular diet. The decreased MDA levels from 0 to 2 h may be attributed to the quickly increased in antioxidant levels between 0 and 2 h of fasting. The quick response of antioxidants following a diet has been seen in some studies (22, 24, 25). The increased trend of MDA levels from 2 to 4 h of fasting may be explained by the continuously increased in lipid oxidation and decreased antioxidant response. Fisher-Wellman et al. (15), found that MDA achieved the highest level at 4 h of fasting following a lipid diet. The decreased antioxidant levels from 1 h of fasting has also been seen in several studies (24, 25).

It has been shown in a previous study that even food intake with low antioxidant levels can increase postprandial total antioxidant activity in older women (22). This is consistent with our study, where there was an increased T-AOC and SOD level between 0 and 2 h. There are several possible explanations for this result. First, diet stimulates the body's antioxidant system to reduce food induced oxidative stress (26). Second, the regular diet contains not only the three major nutrients (carbohydrates, lipids, and proteins), but also minerals and trace elements, which raise the body's antioxidant capacity (18, 24, 25). Finally, as the fasting time increases, the body's antioxidant system defenses restore their usual function and the level of postprandial oxidative stress gradually decreases. Hence the body's T-AOC and SOD level gradually decreases.

This study confirms that diet can induce oxidative stress in the human body, especially lipid oxidation damage. An antioxidant enriched diet may be useful to prevent the oxidation damage induced by diet. It is recommended to fast for at least 8 h to accurately measure lipid oxidation markers (i.e., MDA), T-AOC, and SOD. Since FlOPs are not impacted by diet, fasting is not necessary for their measurement.

This research examined both oxidative stress and antioxidant biomarkers in healthy individuals following a regular diet. The participants were matched by age. Several limitations are acknowledged in the present study. First, the sample size is small. Second, our research only included younger individuals with a limited age range. Age is a well-known risk factor to influence oxidative stress (27–29). Our study results may be different if study the participants of other age range (i.e., the elderly). Third, our observation time was only 10 h following consumption of a meal. We were unable to observe the participants beyond 10 h, because the ethical committee members in our department did not approve longer fasting times due to its potential harmful effects on the human body. Whether longer times will attenuate the results remain unclear.

Postprandial MDA, T-AOC, and SOD decreased with longer fasting times, but FlOPs were not affected by diet. This research adds to the existing evidence on the impact of regular diet on oxidative stress. Further studies with a larger sample size and a longer fasting time are still warranted.

## Data availability statement

The datasets generated and/or analyzed during the current study are not publicly available due to ethical reasons but are available from the corresponding author on reasonable request.

## Ethics statement

The studies involving human participants were reviewed and approved by Institute of Research Board of the School of Public Health, Jilin University (Project#:2018-03-05 and project#:2021-12-06). The patients/participants provided their written informed consent to participate in this study. Written informed consent was obtained from the individual(s) for the publication of any potentially identifiable images or data included in this article.

## Author contributions

SY contributed to the study design and managed the overall project. FH and YZ performed the experiments. FH, XS, and YZ analyzed the data. FH and XS drafted the manuscript. SY and AV amended the manuscript. All authors commented on the manuscript and approved the final version of the manuscript.

## Funding

This work was partly supported by research grants from the Changchun Scientific and Technological Development Program (Grant Numbers: 21ZGM27 and 21ZGM28).

## Conflict of interest

The authors declare that the research was conducted in the absence of any commercial or financial relationships that could be construed as a potential conflict of interest.

## Publisher's note

All claims expressed in this article are solely those of the authors and do not necessarily represent those of their affiliated organizations, or those of the publisher, the editors and the reviewers. Any product that may be evaluated in this article, or claim that may be made by its manufacturer, is not guaranteed or endorsed by the publisher.
